# AI for Poststroke Epilepsy: Scoping Review of Imaging-Based Prediction Models and Implications for African Settings

**DOI:** 10.2196/96828

**Published:** 2026-10-16

**Authors:** Bashemera Brenda Birungi, Rahman Sanya, Suzan Nakasendwa, Joyce Nakatumba Nabende

**Affiliations:** 1 Department of Computer Science, College of Computing and Information Sciences, Makerere University, Hill Road, Kampala, 8HJC+H76, Uganda, 256 756542231; 2 Department of Adult and Community Education, Makerere University, Kampala, Uganda; 3 School of Public Health, Makerere University, Kampala, Uganda

**Keywords:** poststroke epilepsy, stroke, AI, machine learning, deep learning, prediction models, neuroimaging, clinical decision support, scoping review, sub-saharan Africa

## Abstract

**Background:**

Poststroke seizures (PSS) and poststroke epilepsy (PSE) are important neurological complications associated with poorer functional recovery, increased mortality, and reduced quality of life after stroke. Neuroimaging plays a central role in identifying patients at increased risk, and recent advances in AI have enabled the development of imaging-based prediction models that may improve individualized risk stratification. However, the current evidence remains fragmented, and the extent to which these approaches are applicable to African health care settings is unclear.

**Objective:**

This scoping review aimed to chart the existing global evidence on the development of imaging-based prediction models that can help in predicting PSS and PSE, especially AI-based ones. The purpose was to look into the imaging techniques, computational algorithms, and validation methodologies used and the representation of African populations.

**Methods:**

This scoping review was performed using the Joanna Briggs Institute methodological framework, and the results were presented based on the Preferred Reporting Items for Systematic Reviews and Meta-Analyses Extension for Scoping Reviews (PRISMA-ScR) framework. Articles searched using databases such as PubMed, Embase, Scopus, Web of Science, IEEE Xplore, and Google Scholar involved the application of computed tomography (CT), magnetic resonance imaging (MRI), angiography imaging techniques, radiomic markers, or multimodal neuroimaging techniques in statistical and AI-based prediction models for seizure-related outcomes after stroke.

**Results:**

Twenty-three studies published between 2005 and 2026 met the eligibility criteria. The evidence demonstrated a clear methodological evolution from conventional imaging-based prediction models to radiomics, machine learning, deep learning, explainable AI, and multimodal approaches. MRI and CT were the predominant imaging modalities, with cortical involvement, lesion location, infarct volume, hemorrhagic transformation, and quantitative radiomic features emerging as the most frequently reported imaging predictors. Most AI models relied on internal validation, while relatively few underwent external validation. The evidence base was concentrated in East Asia, Europe, and North America, with no eligible imaging-based AI models having been developed or externally validated using African datasets.

**Conclusions:**

Imaging-based AI for predicting PSS and PSE has progressed rapidly, but important methodological and geographical gaps remain. Limited external validation, heterogeneous reporting, and the absence of African development and validation cohorts restrict confidence in the generalizability of existing models. Future research should prioritize the development of explainable imaging-based AI models using representative African neuroimaging datasets and their external validation in multicenter studies.

## Introduction

Stroke remains one of the leading causes of mortality and long-term neurological disability worldwide and is increasingly recognized as the most common cause of acquired epilepsy in adults [1,2]. Improvements in acute stroke care, including reperfusion therapies, specialized stroke units, and advances in secondary prevention, have substantially increased survival following both ischemic and hemorrhagic stroke [3]. Consequently, the number of individuals living with chronic neurological sequelae after stroke has risen considerably. Among these complications, poststroke seizures (PSS) and poststroke epilepsy (PSE) are particularly important because they are associated with increased mortality, poorer functional recovery, cognitive decline, recurrent hospitalizations, and reduced quality of life [4,5]. Beyond their clinical consequences, these conditions place a substantial economic burden on patients, caregivers, and health care systems, particularly in settings with limited neurological services.

PSE, commonly defined as recurrent unprovoked seizures occurring more than 7 days after a stroke, represents one of the leading causes of acquired epilepsy in older adults and accounts for a substantial proportion of new-onset epilepsy worldwide [6]. Although estimates vary according to stroke subtype, population characteristics, and follow-up duration, approximately 5%‐15% of stroke survivors eventually develop epilepsy [5]. However, seizure occurrence after stroke is highly heterogeneous. Some patients develop acute symptomatic seizures shortly after the vascular event, whereas others experience unprovoked seizures months or years later. This variability suggests that epileptogenesis after stroke is a multifactorial and dynamic process that cannot be adequately explained by a single clinical or radiological characteristic.

The mechanisms underlying PSE extend far beyond the initial vascular occurrence. Stroke initiates a cascade of structural and molecular changes, including neuronal death, blood-brain barrier disruption, neuroinflammation, gliosis, synaptic reorganization, and alterations in neuronal excitability, all of which contribute to the development of epileptogenic networks [4,7]. Although several risk factors for PSE have been identified, including stroke severity, cortical involvement, and hemorrhagic transformation, their predictive value differs across studies and populations. Consequently, accurately identifying patients who are likely to develop seizures after stroke remains a major clinical challenge.

Current approaches to seizure risk prediction rely predominantly on clinical prediction scores such as the severity of stroke, large-artery atherosclerotic etiology, early seizures, cortical involvement, and the territory of the middle cerebral artery involvement (SeLECT) score for ischemic stroke and the cortical involvement, age, volume, and early seizures (CAVE) score for intracerebral hemorrhage (ICH) [8,9]. These tools are attractive because they use variables that are routinely available in clinical practice and can easily be implemented at the bedside. Nevertheless, they rely on a relatively small number of manually selected predictors and assume relatively simple relationships between predictors and outcomes. While these models have demonstrated reasonable predictive performance, subsequent studies have shown variability in their performance across populations and health care settings, suggesting that they may not fully capture the biological complexity and heterogeneity of PSE [10]. This has generated increasing interest in more sophisticated predictive approaches capable of leveraging larger and more complex datasets.

Neuroimaging occupies a central role in understanding seizure development after stroke because many of the structural changes of epileptogenesis can be visualized on brain imaging. Across studies, imaging characteristics such as cortical involvement, lesion location, lesion volume, hemorrhagic transformation, temporal lobe injury, and white matter abnormalities have consistently been associated with an increased risk of subsequent seizures and epilepsy [4,11]. However, studies differ considerably in the relative importance assigned to these imaging features and in how they are quantified and incorporated into prediction models. Consequently, there is little consensus regarding which imaging biomarkers are most informative for predicting seizure outcomes across diverse patient populations.

Historically, imaging information has been incorporated into prediction models in a relatively simplistic manner. Most studies have relied on manually derived descriptors, such as the presence or absence of cortical involvement or broad classifications of lesion size and location. Such approaches inevitably reduce the rich information contained within medical images to a limited number of predefined variables and may overlook subtle imaging characteristics associated with epileptogenesis. Moreover, manual interpretation of neuroimaging is subject to interobserver variability and may fail to identify complex patterns that are difficult to detect visually. These limitations have motivated the development of computational methods capable of extracting more comprehensive information from neuroimaging data.

Recent advances in AI have created new opportunities for exploiting the wealth of information embedded within neuroimaging data. Increasing computational power and the growing availability of digital imaging repositories have facilitated the development of imaging-based prediction models capable of extracting high-dimensional information directly from computed tomography (CT) and magnetic resonance imaging (MRI) scans [12,13]. Unlike traditional statistical approaches, AI methods can model complex and nonlinear relationships between imaging characteristics and clinical outcomes, potentially enabling the identification of imaging signatures associated with seizure risk that are not detectable through conventional visual assessment.

Several classes of imaging-based predictive models have emerged over the past decade. Machine learning (ML) algorithms, including support vector machines (SVMs), random forests (RFs), and gradient boosting techniques, have been applied to imaging-derived data to model complex relationships between predictors and seizure outcomes. More recently, radiomics approaches have enabled the extraction of large numbers of quantitative imaging features describing lesion shape, texture, intensity, and spatial heterogeneity [14,15]. These radiomic features are subsequently incorporated into ML algorithms to generate predictive models of PSE. In parallel, deep learning (DL) approaches, particularly convolutional neural networks (CNNs), have increasingly been applied to neuroimaging data because they can automatically learn hierarchical representations directly from raw images without requiring manual feature engineering [16,17].

Despite these developments, the literature on imaging-based prediction of PSS and PSE remains fragmented. Existing studies differ substantially in their objectives, imaging modalities, modeling approaches, and definitions of outcomes. Some studies focus on predicting acute symptomatic seizures occurring shortly after stroke, whereas others aim to estimate the long-term risk of epilepsy developing months or years after the initial event. Similarly, considerable heterogeneity exists in the imaging modalities used, ranging from conventional CT scans and structural MRI sequences to advanced radiomic features derived from these modalities. This heterogeneity makes direct comparison of findings difficult and complicates efforts to identify the most promising approaches for clinical implementation.

The methods used to develop prediction models are equally diverse. Studies have used a broad spectrum of algorithms, including logistic regression models incorporating imaging variables, SVMs, RFs, gradient boosting techniques, radiomics-based classifiers, and DL models. However, the evaluation of these models has generally lagged behind their development. Although many studies report favorable predictive performance, most rely exclusively on internal validation strategies such as split-sample testing and cross-validation. External validation using independent datasets remains uncommon, limiting confidence in the robustness and transportability of existing models [18]. Furthermore, evidence regarding whether increasingly sophisticated approaches, such as DL, consistently outperform conventional ML methods remains limited.

Another notable feature of the literature is its uneven geographical distribution. The overwhelming majority of imaging-based prediction models for PSS and PSE have been developed using datasets from Europe, North America, and East Asia, where access to advanced neuroimaging infrastructure, digital health systems, and computational resources is relatively well established. In contrast, evidence from countries with limited resources remains extremely limited. This underrepresentation is particularly concerning in Africa, where the burden of stroke is increasing rapidly and is associated with disproportionately high mortality and disability [19,20]. Furthermore, many health care systems in the region rely predominantly on CT imaging because MRI remains inaccessible in many settings, and access to specialist neurological services is often limited.

The absence of African populations in the development and validation of imaging-based prediction models raises important concerns regarding the applicability of existing tools within these settings. Differences in stroke epidemiology, imaging acquisition protocols, health care pathways, and patient demographics may lead to substantial dataset shift, whereby models developed in one population perform poorly when applied to another [21]. In addition, algorithms trained predominantly on populations from high-income countries may inadvertently introduce bias and perpetuate disparities if implemented without adequate local validation. At the same time, increasing digitization of radiology services and growing interest in AI applications across African health care systems provide important opportunities for the development of contextually appropriate prediction models. CT-based approaches may be particularly attractive because CT remains the most widely available imaging modality for acute stroke assessment in many low-resource settings.

Although several reviews have examined PSS and PSE from epidemiological and clinical perspectives, none has systematically mapped the rapidly growing literature on imaging-based prediction models or critically examined their methodological characteristics, validation strategies, and relevance to African health care settings. Consequently, there remains limited understanding of the global landscape of imaging-based predictive modeling for PSE and of the evidence gaps that must be addressed before these approaches can be translated to underrepresented populations. Therefore, this scoping review aims to map the global evidence on imaging-based prediction models for PSS and PSE, with particular emphasis on their applicability to African settings. Specifically, this review addresses the following research questions:

1. What imaging-based prediction models have been developed globally for PSS and PSE?
2. What AI methods, imaging modalities, and validation strategies have been used?
3. To what extent are African populations represented in these studies?
4. What barriers and opportunities exist for translating these models to African health care settings?

This scoping review seeks to provide a comprehensive overview of current imaging-based predictive approaches and to identify critical evidence gaps that must be addressed to enable the development of equitable, clinically relevant, and contextually appropriate prediction models for PSE in Africa and other limited resource settings.

## Methods

### Overview

This scoping review was performed to systematically identify, map, and synthesize the global evidence on imaging-based prediction models, including conventional statistical and AI-based approaches, for predicting PSS and PSE. In addition to characterizing the methodological landscape of existing prediction models, the review sought to examine the extent to which African populations have been represented in model development and validation and to identify opportunities and challenges for translating these approaches to African health care settings. Given the methodological diversity of the available literature, including differences in imaging modalities, feature engineering strategies, AI algorithms, prediction targets, and validation approaches, a scoping review was considered the most appropriate methodology for comprehensively mapping the evidence base and identifying knowledge gaps rather than evaluating the comparative effectiveness of individual prediction models.

### Study Design

The review was conducted in accordance with the methodological framework for scoping reviews originally proposed by Arksey and O’Malley [22] and further refined by Levac et al [23] and the Joanna Briggs Institute (JBI) manual for evidence synthesis [24]. These frameworks recommend a structured and iterative approach to evidence mapping that is particularly suited to emerging research areas characterized by methodological diversity and evolving concepts. The review was reported in accordance with the Preferred Reporting Items for Systematic Reviews and Meta-Analyses Extension for Scoping Reviews (PRISMA-ScR) guidelines [25], which provides a standardized framework for transparent and reproducible reporting of scoping reviews.

A scoping review methodology was selected because imaging-based AI for predicting seizure-related outcomes after stroke represents a rapidly evolving field spanning neurology, neuroradiology, medical image analysis, computer vision, and AI. Studies in this area differ substantially in their prediction objectives, imaging modalities, feature extraction techniques, AI algorithms, validation procedures, and reporting standards. Such methodological heterogeneity limits the feasibility and interpretability of quantitative evidence synthesis and instead necessitates an approach capable of systematically mapping the breadth of available evidence.

Furthermore, the objectives of this review extend beyond identifying existing predictive models to understanding how these models have been developed, validated, and reported, while evaluating their potential applicability to African health care settings. Consequently, the review emphasizes methodological characteristics, geographical representation, and translational considerations rather than comparative assessment of model performance. This approach is consistent with the primary purpose of scoping reviews, namely, to characterize the extent, nature, and distribution of available evidence while identifying gaps that warrant further investigation.

### Protocol Registration

The review protocol was prospectively registered on the Open Science Framework (OSF) before study selection [26,27]. It prespecified the review objectives, eligibility criteria, search strategy, screening process, data-charting framework, and evidence synthesis methods to ensure transparency and reproducibility. During study screening, no eligible imaging-based AI models that had been developed or externally validated using African datasets were identified. Consequently, the review was reframed from an Africa-focused synthesis to a global mapping of imaging-based prediction models with emphasis on their implications for African health care settings. This refinement ensured that the review reflected the available evidence while maintaining its objective of informing future AI development and implementation in underrepresented populations.

Following preliminary screening, the scope of the evidence synthesis was refined to include conventional imaging-based statistical prediction models alongside AI-based approaches. This refinement allowed the review to map the methodological evolution from conventional imaging-derived prediction to radiomics, ML, and DL while retaining the predefined population, imaging, and seizure-outcome criteria. The overarching objective of evaluating the methodological landscape and its relevance to African health care settings remained unchanged.

### Eligibility Criteria

#### Overview

Eligibility criteria were established a priori using the population-concept-context (PCC) framework recommended by the JBI for scoping reviews [24]. The PCC framework was selected because it provides a flexible yet systematic approach for defining study eligibility in reviews that seek to map heterogeneous bodies of evidence rather than evaluate intervention effectiveness. In the present review, the framework facilitated transparent identification of studies relevant to imaging-based AI for PSS and PSE prediction while ensuring consistency throughout the screening and selection process. The detailed eligibility criteria and operational definitions used during study screening are provided in Tables 1 and 2.

The detailed eligibility criteria and operational definitions presented in Tables 1 and 2 were used consistently throughout title and abstract screening, full-text assessment, and data charting to ensure methodological transparency, reproducibility, and conceptual consistency during evidence synthesis.

**Table 1.** Eligibility criteria based on the PCC^a^ framework.

Framework	Inclusion criteria	Exclusion criteria
Population	Adults aged ≥18 years with ischemic stroke, intracerebral hemorrhage, or mixed stroke populations investigated for early poststroke seizures, late poststroke seizures, or poststroke epilepsy.	Pediatric populations, epilepsy unrelated to stroke, traumatic brain injury, brain tumors, central nervous system infections, animal studies, or other neurological conditions unrelated to stroke.
Concept	Imaging-based prediction models using CT^b^, MRI^c^, angiographic imaging, radiomics, or multimodal neuroimaging to predict PSS^d^ or PSE^e^. Eligible approaches included conventional statistical prediction models when imaging-derived variables contributed to prediction, machine learning, radiomics, and deep learning.	Studies relying solely on clinical, demographic, laboratory, EEG^f^, or electronic health record variables without imaging-derived information; studies that did not develop or evaluate prediction of PSS/PSE.
Context	Studies conducted globally, with geographical representation assessed and particular attention to African populations and implications for African health care settings.	Studies unrelated to stroke or without an imaging/imaging-derived component.
Publication	Original peer-reviewed research articles published in English; no restriction on publication year.	Reviews, editorials, conference abstracts without full-text publications, letters, protocols, dissertations, case reports/case series, book chapters, and other nonoriginal research publications.

a PCC: population-concept-context.

b CT: computed tomography.

c MRI: magnetic resonance imaging.

d PSS: poststroke seizures.

e PSE: poststroke epilepsy.

f EEG: electroencephalography.

**Table 2.** Operational definitions adopted during study screening and evidence synthesis.

Term	Operational definition
Early PSS^a^	Seizure occurring within 7 days of stroke onset.
Late PSS	Seizure occurring more than 7 days after stroke onset.
PSE^b^	Recurrent unprovoked seizures occurring after stroke, consistent with International League Against Epilepsy recommendations.
Imaging-based prediction model	A predictive model in which CT^c^, MRI^d^, angiographic imaging, radiomic features, or other neuroimaging-derived variables contribute to prediction of early PSS, late PSS, or PSE.
Conventional statistical prediction model	Logistic regression, Cox proportional hazards regression, nomograms, clinical risk scores, or related statistical models in which imaging-derived variables contribute to prediction. These were treated as an eligible evidence category but were not classified as AI.
Imaging-based AI	Computational approaches that learn predictive relationships from neuroimaging-derived data rather than relying solely on prespecified statistical assumptions.
Machine learning	Algorithms applied primarily to manually engineered imaging-derived features, including support vector machines, random forests, decision trees, gradient boosting, extreme gradient boosting, k-nearest neighbors, ensemble methods, and artificial neural networks.
Radiomics	Quantitative extraction of imaging features such as intensity, texture, morphology, and higher-order spatial characteristics from CT or MRI, followed by predictive modeling.
Deep learning	Neural-network architectures, including CNNs^e^, RNNs^f^, transformers, hybrid neural networks, and related approaches that automatically learn hierarchical representations from imaging data.
African setting	Studies conducted in one or more African countries, including patient populations in Africa, or explicitly addressing the development, validation, implementation, or deployment of prediction models within African health care systems.
Validation strategy	The approach used to evaluate model performance, including internal validation such as cross-validation or split-sample validation, temporal validation, or external validation using an independent dataset.
Prediction outcome	Prediction of early PSS, late PSS, or PSE.

a PSS: poststroke seizures.

b PSE: poststroke epilepsy.

c CT: computed tomography.

d MRI: magnetic resonance imaging.

e CNN: convolutional neural network.

f RNN: recurrent neural network.

#### Population

The review considered studies involving adult patients (aged ≥18 years) diagnosed with ischemic stroke, ICH, or mixed stroke populations in whom seizure-related outcomes following stroke were investigated. Eligible outcomes included early PSS, late PSS, and PSE, reflecting the continuum of epileptogenesis following cerebrovascular injury [7,28]. For consistency during study selection, early PSS were defined as seizures occurring within 7 days of stroke onset, whereas late PSS referred to seizures occurring more than 7 days after stroke. PSE was defined as recurrent unprovoked seizures occurring after stroke in accordance with contemporary International League Against Epilepsy (ILAE) recommendations [29]. Studies involving pediatric populations, epilepsy unrelated to stroke, traumatic brain injury, brain tumors, central nervous system infections, or other neurological disorders were excluded because their underlying pathophysiological mechanisms, neuroimaging characteristics, and predictive risk factors differ substantially from those associated with stroke.

#### Concept

Imaging-based prediction of PSS was the main topic. Eligible studies were required to develop or evaluate an imaging-based prediction model whereby neuroimaging or imaging-derived markers were used to predict early PSS, late PSS, or PSE. Imaging modalities eligible for inclusion in this review included CT, MRI, angiographic imaging, radiomics, and multimodal neuroimaging. Conventional statistical models as well as AI methods were considered for the purpose of charting the methodological development of the imaging-based prediction process.

Prediction models were categorized into 4 broad categories for evidence synthesis. Conventional statistical imaging-based prediction models included logistic regression, Cox proportional hazards regression, nomograms, and clinical risk scores when imaging-derived variables served as components of the prediction models [30-32]. ML involved supervised learning techniques applied to imaging-derived features, including SVMs, RF, DTs, gradient boosting, extreme gradient boosting (XGBoost), k-nearest neighbors, ensemble, and artificial neural networks [33]. Radiomics involved feature extraction from either CT or MRI, which was quantitative in nature and included intensity, texture, morphology, and high-order spatial features followed by application of predictive modeling via ML [34]. DL involved CNNs, recurrent neural networks (RNNs), transformers, hybrid neural networks, and other neural network architectures that learned hierarchical representations from images [35].

In this review, the operational definition of AI included computational methods that learned predictive relationships from data rather than making a priori statistical assumptions about relationships [36]. Traditional statistical modeling was thus excluded as a form of AI but remained an eligible evidence category because it provided a basis for evaluating imaging biomarkers and prediction models developed using AI methods. Evidence generated purely from clinical factors, demographics, laboratory factors, electroencephalography (EEG) factors, electronic health records, or other nonimaging factors was also excluded from the evidence pool. Logistic regression, Cox proportional hazards regression, nomograms, and clinical risk scores were only eligible if imaging-related factors contributed to the prediction model [30-32].

#### Context

Studies conducted in all geographical regions were eligible for inclusion. Although the review specifically sought to evaluate the implications of imaging-based AI for African health care settings, restricting eligibility to African studies alone would have precluded meaningful synthesis because preliminary screening identified no eligible imaging-based AI prediction models developed or externally validated using African datasets. Consequently, global studies were intentionally included to comprehensively map the current methodological landscape, evaluate geographical representation within existing evidence, and identify methodological and translational gaps relevant to future research and implementation in African health care systems.

For the purposes of this review, a study was considered to represent an African setting if patient data were collected within one or more African countries, African populations formed part of the study cohort, or the study explicitly discussed development, validation, implementation, or deployment of predictive models within African health care systems. Only original peer-reviewed research articles published in English were included. Reviews, editorials, conference abstracts without full-text publications, letters, study protocols, case reports, case series, dissertations, book chapters, and animal studies were excluded because they did not provide sufficient methodological detail for data extraction and evidence synthesis.

### Information Sources

A comprehensive literature search was conducted to identify published studies describing imaging-based prediction models, including conventional statistical and AI-based approaches**,** for predicting PSS and PSE. Given the multidisciplinary nature of this research area, databases were selected to ensure broad coverage of the clinical, neuroimaging, and computational literature. PubMed/MEDLINE and Embase were searched because these databases provide extensive coverage of biomedical research, including neurology, stroke medicine, neuroradiology, and epilepsy [37,38]. Scopus and Web of Science were included to capture interdisciplinary studies published across the medical, engineering, and computer science literature while also facilitating citation tracking and identification of highly cited publications [39,40].

IEEE Xplore was searched to identify methodological studies originating from the AI, computer vision, and medical image analysis communities, where advances in imaging-based predictive modeling are frequently first reported [41]. To minimize publication bias and maximize retrieval of relevant evidence, Google Scholar was additionally searched to identify potentially eligible studies that may not yet have been indexed within the primary bibliographic databases [42]. Citation chasing was also undertaken by manually screening the reference lists of all included studies and relevant review articles, while forward citation tracking was performed where appropriate to identify recently published articles citing the included studies.

The electronic search was initially conducted on September 1, 2025, and updated on July 7, 2026, prior to final analysis to ensure inclusion of the most recent eligible studies. No restrictions were placed on publication year because the objective of the review was to comprehensively map the evolution of imaging-based AI approaches for seizure prediction following stroke. Only studies published in English were considered eligible because of resource limitations associated with translation and the need to ensure accurate interpretation of technical descriptions relating to AI methodologies.

### Search Strategy

The search strategy was developed around four concept domains: (1) stroke, (2) PSS and PSE, (3) neuroimaging, and (4) prediction modeling. Synonymous terms within each concept were combined using the Boolean operator OR, while the 4 concept domains were combined using AND. Quotation marks were used for multiword phrases where supported by the respective database, and truncation and database-specific field tags were applied where appropriate. The search strategy was adapted for PubMed/MEDLINE, Embase, Scopus, Web of Science, IEEE Xplore, and Google Scholar. No publication-year restriction was applied. English-language restrictions were applied using database filters and during screening. Complete database-specific search strings, including all keywords, controlled vocabulary where applicable, Boolean operators, quotation marks, filters, and numbers of records retrieved, are provided in Multimedia Appendix 1.

### Study Selection

Following completion of the electronic searches, all retrieved records were imported into Zotero (Corporation for Digital Scholarship) for reference management. Duplicate records were identified using the software’s automated duplicate-detection function and were subsequently verified through manual inspection to ensure accurate removal.

Study selection was undertaken in 2 sequential stages. During the first stage, 2 reviewers (BBB and SN) independently screened titles and abstracts against the predefined eligibility criteria. Articles considered potentially relevant by either reviewer progressed to full-text assessment to minimize the risk of excluding eligible studies prematurely. During the second stage, full-text articles were independently assessed by the same reviewers to determine final eligibility. Studies were included only if they satisfied all predefined PCC eligibility criteria. Reasons for exclusion at the full-text stage were documented to ensure transparency and reproducibility of the selection process.

Prior to formal screening, the reviewers independently screened a pilot sample of retrieved studies to ensure consistent interpretation of the eligibility criteria and operational definitions adopted in this review. Any uncertainties arising during the pilot phase were discussed, and the screening criteria were refined where necessary before commencement of the full review. Disagreements arising during title and abstract screening, full-text assessment, or data extraction were resolved through discussion and consensus. Where consensus could not be reached, a third reviewer was available to adjudicate disagreements. The study selection process is summarized in a PRISMA-ScR flow diagram.

### Data Charting

#### Data Overview

Data charting was conducted using a standardized data extraction form developed specifically for this review in accordance with recommendations from the JBI for scoping reviews. The charting framework was designed to systematically capture methodological, technical, and contextual characteristics of imaging-based prediction models while remaining sufficiently flexible to accommodate the diversity of study designs encountered during the review. The data charting form was initially piloted using a subset of included studies to evaluate the clarity, completeness, and consistency of the extraction fields.

Following the pilot exercise, minor refinements were made to improve consistency in recording imaging modalities, AI methodologies, validation approaches, and outcome definitions. The finalized charting form was then applied to all included studies. Data extraction was performed independently by 2 reviewers. Throughout the charting process, regular discussions were held to resolve uncertainties and ensure consistent interpretation of extracted variables. Any discrepancies identified during data extraction were resolved through consensus, with consultation from a third reviewer where necessary.

Consistent with scoping review methodology, the charting process remained iterative throughout the review. Where additional variables emerged as important for addressing the review objectives, particularly those relating to African representation, model implementation, and methodological transparency, these variables were incorporated into the charting framework and retrospectively extracted from previously reviewed studies where applicable.

#### Data Items

Data extraction was guided by the review objectives to characterize the methodological landscape of imaging-based prediction models for PSS and PSE, with particular emphasis on the transition from conventional statistical approaches to AI-based methods. For each included study, bibliographic information (first author, publication year, and country), study design, data source, sample size, patient population, stroke subtype, and seizure-related outcomes were extracted. Technical data included imaging modality (CT, MRI, or multimodal imaging), preprocessing methods, feature extraction, AI approach, model architecture, prediction target, explainability techniques, and validation strategy (internal, temporal, or external).

To assess the relevance of existing models to African health care settings, information on geographical representation, African populations, imaging and computational requirements, implementation considerations, reported limitations, dataset shift, imaging variability, algorithmic bias, and deployment in resource-constrained settings was extracted where available.

Model performance metrics, including the area under the receiver operating characteristic curve (AUC), sensitivity, specificity, accuracy, precision, recall, *F*_1_-score, calibration measures, and other relevant evaluation metrics, were extracted where available [43]. These metrics were synthesized narratively rather than pooled quantitatively to compare predictive performance across methodological approaches.

#### Data Quality Appraisal

Consistent with the objectives of a scoping review and current JBI guidance, a formal methodological quality assessment or risk-of-bias appraisal was not undertaken because the purpose of the review was to map the breadth, characteristics, and methodological trends of the available evidence rather than evaluate the comparative effectiveness or certainty of individual studies.

#### Data Availability

All data generated or analyzed during this study are included in this published article and its supplementary materials. The study screening workbook, data charting table, and PRISMA-ScR checklist are provided as Checklist 1.

### Ethical Considerations

Ethics approval was not required because this study synthesized data from published literature and did not involve human participants, patient-level data, or identifiable information.

## Results

### Overview

The results of this scoping review are presented descriptively, mapping the characteristics, methodological approaches, and outcomes of the included studies in accordance with PRISMA-ScR guidance.

### Study Selection

A total of 86 references were identified from the database searches. Following exclusion of 15 duplicate studies, 71 references were subjected to title/abstract screening, of which 56 were excluded. Fifteen studies identified from the database searches underwent full-text screening. An additional 8 studies identified through citation searching were included. Thus, 23 studies met the inclusion criteria [8,10,44-64].

The final evidence synthesis included studies describing conventional imaging-based prediction models, radiomics-based models, ML approaches, DL methods, and computational lesion-mapping techniques. The included studies collectively represent the current global evidence on imaging-informed prediction of PSS and PSE while highlighting the limited translation of these approaches to African health care settings. A detailed overview of the study selection process is presented in the PRISMA-ScR flow diagram shown in Figure 1.

**Figure 1.**
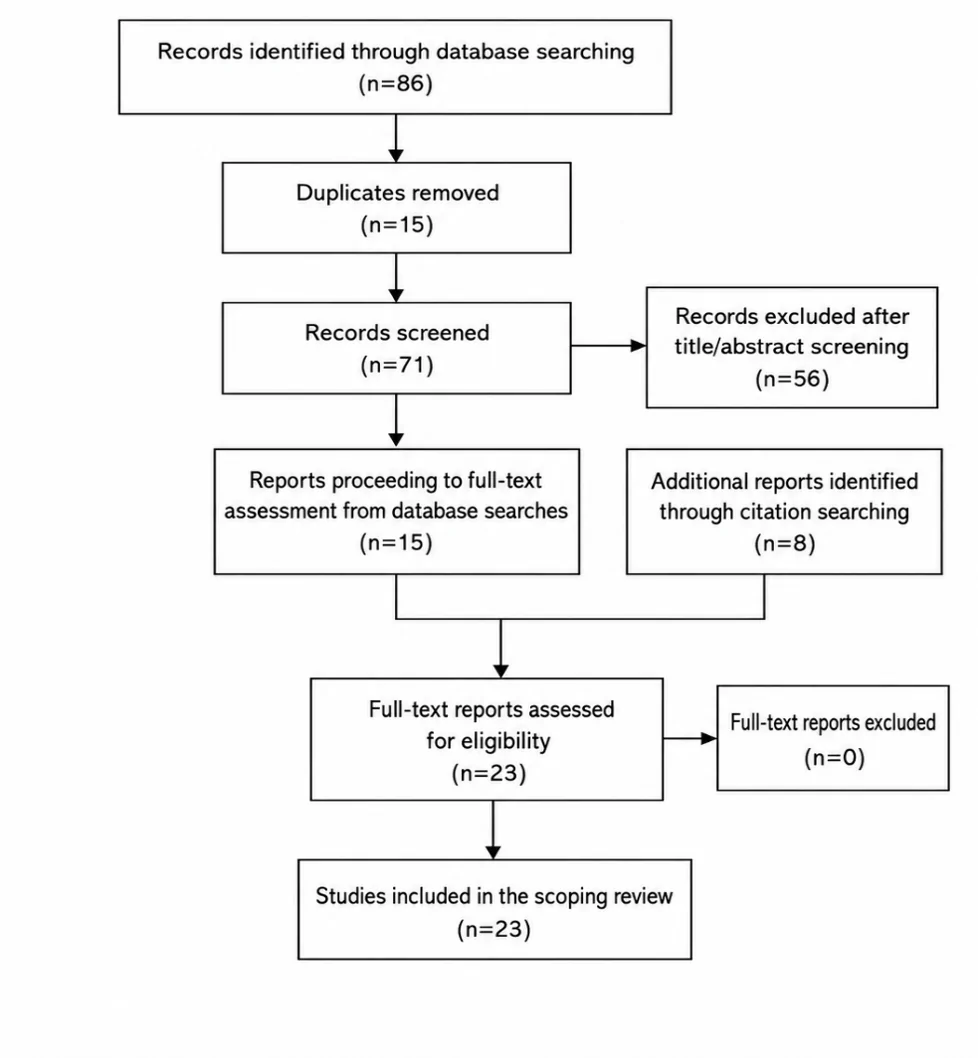
PRISMA-ScR (Preferred Reporting Items for Systematic Reviews and Meta-Analyses Extension for Scoping Reviews) flow diagram illustrating study identification, screening, full-text eligibility assessment, and final inclusion.

### Characteristics of Included Studies

The included studies, published between 2005 and 2026, demonstrate the evolution of imaging-based prediction from conventional statistical models to advanced AI approaches. Early studies focused on imaging biomarkers such as lesion size, cortical involvement, infarct location, hemorrhagic transformation, and stroke severity using multivariable regression models [44-46]. More recent investigations incorporated radiomics, ML, DL, and explainable AI to improve individualized prediction of seizure risk after stroke [47-49].

Sample sizes ranged from fewer than 100 participants in exploratory imaging studies to more than 21,000 patients in multicenter ML investigations [50]. Conventional studies generally enrolled 200‐1500 participants, whereas externally validated AI models increasingly used multicenter cohorts exceeding 1000 patients [47,49]. Follow-up periods varied from 6 months to more than 7 years according to study objectives.

Most studies investigated acute ischemic stroke, particularly patients undergoing mechanical thrombectomy for anterior circulation large-vessel occlusion [50-52]. Fewer studies examined ICH, although these contributed substantially to advances in radiomics and DL [47,48], while some included mixed stroke populations [53].

MRI was the predominant imaging modality, particularly diffusion-weighted, transverse relaxation time–weighted (T2-weighted), and postthrombectomy MRI, providing measures of infarct volume, lesion topology, cortical involvement, cerebral microbleeds, enlarged perivascular spaces, and voxel-based lesion maps [52,54,55]. CT remained the primary modality for hemorrhagic stroke, supporting both conventional imaging assessment and radiomic feature extraction, whereas angiographic imaging provided prognostic information through collateral circulation, early venous filling, and angiographic blush signs [44]. Methodologically, the evidence progressed from logistic and Cox regression models identifying imaging predictors of PSE [44,46,56] to imaging-based risk scores such as SeLECT, hemorrhagic transformation, severity of stroke, large-artery atherosclerotic etiology (H-SeLECT), and infarct size, cortical involvement, hemorrhagic transformation, early seizures, middle cerebral artery involvement, and age younger than 65 years (IsCHEMiA) [10,47,65], before advancing to radiomics, CNNs, explainable ML, and voxel-based lesion mapping for automated identification of imaging patterns associated with PSE [45,48,49].

Overall, imaging-based prediction of PSE has advanced considerably, but development has largely relied on retrospective datasets from well-resourced health care systems. No included study developed or externally validated a model using African imaging cohorts, highlighting a major geographical evidence gap and an important priority for future research. All these details are summarized in Table 3.

**Table 3.** Characteristics, performance, and validation of the included studies.

Study	Country/region	Design, sample, and stroke subtype	Imaging and method	Outcome/time window	Main performance measure	Validation
Wang et al (2025) [48]	China	Retrospective single-center n=1130 ICH^a^	Noncontrast CT^b^ PSENet^c^ deep learning	PSE^d^ follow-up as reported	AUC^e^ 0.840 (95% CI 0.792‐0.898) accuracy 0.876 *F*_1_-score: 0.621	5-fold cross-validation; no independent external cohort
Lin et al (2023) [57]	China	Retrospective 2-center; n=1571; first-ever ICH	CT radiomics plus clinical model	PSE; study-defined follow-up	Combined model AUC 0.85 (95% CI 0.78‐0.92); clinical model AUC 0.81	Derivation and independent validation cohorts from 2 tertiary hospitals
Wang et al (2026) [50]	China	Retrospective; n=365; ischemic/hemorrhagic stroke	EEG^f^-fMRI^g^ plus clinical biomarkers; RF^h^/SVM^i^/GB^j^	PSE; study-defined follow-up	Validation AUCs: RF 0.731, SVM 0.746, GB 0.811; Brier: 0.067, 0.083, and 0.075	Random 70:30 split; no external cohort
Dasari et al (2026) [58]	United States	Retrospective; n=221; acute ischemic stroke	DWI^k^ MRI^l^; CNN^m^-derived infarct volume plus Cox modeling	PSE; time-to-event follow-up	C-index^n^/AUC not consistently reported in manuscript source; association estimates reported	Internal modeling with calibration; no external cohort
Gruber et al (2025) [59]	Austria	Bicenter retrospective; n=251; LVO^o^ stroke after thrombectomy	Postinterventional MRI; voxel-based lesion-symptom mapping	PSE; longitudinal follow-up	Discrimination metric not reported; lesion-location associations reported	Internal bicenter analysis
Leung et al (2026) [47]	United States, Hong Kong, Japan	Development n=1436; external validation n=2534; ischemic stroke	CT/MRI-derived features; IsCHEMiA^z^ competing-risk score	PSE; 1- and 5-year risk	Overall c-statistic 0.848 vs 0.782 for SeLECT^aa^	3 independent international external cohorts; calibration assessed
Chou et al (2022) [54]	Taiwan	Retrospective case-control; n=132; cerebral infarction	MRI lesion maps; voxel/volumetric analysis	Early PSS^p^ (≤7 days) and late PSS (>7 days)	AUC/C-index not reported; strategic lesion-location associations reported	Internal matched analysis
Gruber et al (2023) [60]	Austria; Germany	Multicenter retrospective; n=348; LVO stroke after thrombectomy	Postinterventional MRI; Cox regression	PSE; longitudinal follow-up	C-index/AUC not reported; adjusted hazard associations reported	Internal multicenter cohort
Takase (2020) [45]	Japan	Retrospective; n=93; cardioembolic infarction	DWI MRI; multivariable logistic regression	PSE; study-defined follow-up	AUC/C-index not reported; parietal lesion association reported	Internal cohort
Thevathasan et al (2018) [61]	Australia	Consecutive cohort; n=205; anterior-circulation stroke after EVT^q^	24-hour CT/MRI; time-to-event regression	PSS; acute and follow-up events	AUC/C-index not reported; hemorrhagic-transformation association reported	Internal cohort
Jiang et al (2025) [51]	China	Multicenter retrospective; n=1167; LVO stroke after thrombectomy	Angiographic collateral grading; competing-risk analyses	PSE; longitudinal follow-up	AUC/C-index not reported in extracted source; adjusted association estimates reported	Internal multicenter cohort and sensitivity analyses
Jiang et al (2026; angiographic signs) [52]	China	Multicenter retrospective; n=800 (301 matched); anterior-circulation stroke	Digital subtraction angiography plus infarct imaging	PSE; longitudinal follow-up	AUC/C-index not reported in extracted source; diagnostic/association measures reported	Internal multicenter cohort
Yu et al (2022) [55]	China; Australia	Retrospective; n=312; acute ischemic stroke	T2^r^ MRI; enlarged perivascular-space asymmetry	PSE; study-defined follow-up	AUC/C-index not reported; multivariable association estimates reported	Internal cohort
Ishihara et al (2024) [62]	Japan	Retrospective; n=237; acute LVO	CT ASPECTS^s^/infarct volume; conventional predictor analysis	Acute symptomatic seizure (≤7 days) and PSE	AUC/C-index not reported; incidence and adjusted associations reported	Internal cohort
Pozo Putalivo et al (2025) [53]	Argentina	Retrospective; n=760; ischemic/hemorrhagic stroke	CT/MRI lesion characteristics; SeLECT/CAVE comparison	PSE; study-defined follow-up	Performance reported for score comparison; exact metric not consistently available in extracted source	Internal cohort
Galovic et al (2018) [8]	Switzerland; Austria; Germany; Italy	Development n=1200; external validation n=1169; ischemic stroke	Clinical and imaging predictors; SeLECT Cox model	Late seizures (>7 days); 1- and 5-year risk	External validation c-statistic 0.77 (95% CI 0.71‐0.82)	3 independent external cohorts; calibration plots
Meletti et al (2024) [10]	Italy	Retrospective; n=1094; first-ever ischemic stroke after reperfusion	Modified SeLECT 2.0 using posttreatment outcome	PSE; 12‐60 months	Improved time-dependent discrimination versus comparator score; exact values reported by horizon	Internal temporal-horizon comparison
Kim et al (2016) [46]	South Korea	Retrospective score derivation; n=124 patients with first PSS	Lesion size/cortical involvement; conventional risk score	Seizure recurrence/PSE; follow-up after first seizure	Sensitivity/specificity reported; AUC/C-index not reported	Internal cohort
Kammersgaard and Olsen (2005) [44]	Denmark	Prospective community cohort; n=1197; mixed stroke	CT lesion size/location; logistic regression	PSE; up to 7 years	AUC/C-index not reported; incidence and predictor estimates reported	Internal long-term cohort
Ebrahimian et al (2024) [63]	Iran	Prospective; n=309; cortical acute ischemic stroke	DWI MRI; DWI-ASPECTS plus logistic regression	Early PSS (<=7 days)	AUC analysis reported; exact value not available in extracted source	Internal cohort
Alet et al (2022) [56]	Argentina	Retrospective; n=691; ischemic stroke	Fazekas score, cortical involvement, hemorrhagic transformation	PSE; study-defined follow-up	AUC/C-index not reported; adjusted association estimates reported	Internal cohort
Liu et al (2024) [64]	China	Multicenter retrospective; n=21,459; acute ischemic stroke	Imaging reports plus clinical/lab features; 9 ML^t^ algorithms and SHAP^u^	PSE within 1 year	Tree models AUC about 0.99; Brier score 0.006; external RF sensitivity 0.91, PPV^v^ 0.95	70:30 split plus external cohort (n=536); SMOTE-ENN^w^ for imbalance
Jiang et al (2026; explainable ML) [49]	China	Multicenter retrospective; development n=1185 plus 3 external cohorts	Procedure/imaging-derived variables; 8 ML algorithms; RSF^x^ and SHAP	PSE at 1, 3, and 5 years	External C-indices 0.812, 0.822, 0.763; AUC ranges 0.748‐0.839	3 independent external cohorts; calibration, integrated Brier score, DCA^y^

a ICH: intracerebral hemorrhage.

b CT: computed tomography.

c PSENet: poststroke epilepsy network.

d PSE: poststroke epilepsy.

e AUC: area under the receiver operating characteristic curve.

f EEG: electroencephalography.

g fMRI: functional magnetic resonance imaging.

h RF: random forest.

i SVM: support vector machine.

j GB: gradient boosting.

k DWI: diffusion-weighted imaging.

l MRI: magnetic resonance imaging.

m CNN: convolutional neural network.

n C-index: concordance index.

o LVO: large-vessel occlusion.

p PSS: poststroke seizure.

q EVT: endovascular thrombectomy.

r T2: transverse relaxation time.

s ASPECTS: Alberta Stroke Program Early CT Score.

t ML: machine learning.

u SHAP: Shapley Additive Explanations.

v PPV: positive predictive value.

w SMOTE-ENN: Synthetic Minority Oversampling Technique–Edited Nearest Neighbors.

x RSF: random survival forest.

y DCA: decision curve analysis.

z IsCHEMiA: infarct size, cortical involvement, hemorrhagic transformation, early seizures, middle cerebral artery involvement, and aged younger than 65 years.

aa SeLECT: severity of stroke, large-artery atherosclerotic etiology, early seizures, cortical involvement, and the territory of the middle cerebral artery involvement.

ab CAVE: cortical involvement, age, volume, and early seizures.

### Geographic Distribution of the Studies

The geographical distribution of the included studies was heavily concentrated in high-income countries, with most evidence originating from East Asia, Europe, and North America (Figures 2 and 3).

China contributed the largest number of studies, particularly in radiomics, DL, and explainable ML for predicting PSE following ICH or mechanical thrombectomy [47-49,52]. Japan, Hong Kong, and the United States also contributed substantially through the development and external validation of imaging-based prediction models, including the internationally validated IsCHEMiA score.

European studies, mainly from Austria, Germany, Denmark, Italy, and Spain, focused on structural imaging biomarkers associated with PSE. These studies [47,53,54] investigated infarct topology, lesion volume, cortical involvement, hemorrhagic transformation, and voxel-based lesion mapping using MRI to improve epilepsy risk stratification. Multicenter European studies also highlighted growing use of imaging-derived prognostic markers following mechanical thrombectomy.

Recent studies increasingly adopted multicenter and internationally validated cohorts, improving model robustness and generalizability. For example, the IsCHEMiA score was externally validated in cohorts from the United States, Hong Kong, and Japan, consistently outperforming earlier prediction scores [47]. However, international validation remained confined to high-income health care systems with comparable imaging infrastructure.

Despite broader geographical representation across Asia, Europe, and North America, no eligible study reported the development, external validation, or prospective evaluation of imaging-based AI models using African neuroimaging datasets. Likewise, no international multicenter study included African hospitals. This absence was consistent across conventional imaging-based risk scores, radiomics, ML, DL, and lesion-mapping approaches.

This geographical imbalance represents a major finding of the review. Although imaging-based prediction has advanced rapidly, progress has been driven almost entirely by well-resourced health care systems with advanced neuroimaging, specialized stroke services, and large imaging repositories. Consequently, evidence on model performance and applicability in African health care settings remains limited, where differences in stroke epidemiology, imaging availability, health care infrastructure, and patient demographics may affect model transportability. This gap highlights the need for multicenter studies to develop and externally validate imaging-based prediction models using representative African stroke populations.

**Figure 2.**
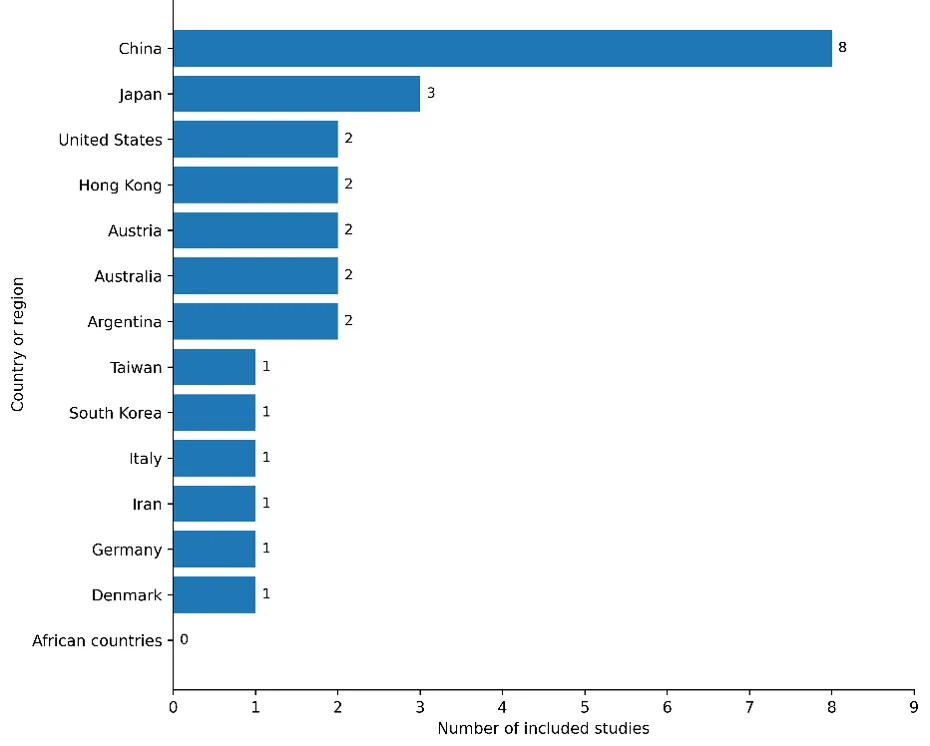
Global distribution of the studies included in the review. Countries participating in multicountry studies were counted individually. No included study used an African development or validation cohort.

### Imaging Modalities Used

Brain imaging underpinned all prediction models included in this review, although imaging modalities and extracted features varied considerably. CT, MRI, angiographic imaging, and quantitative radiomic features were the principal imaging sources used to predict PSS and PSE.

MRI was the most frequently used modality. Diffusion-weighted imaging (DWI), T2-weighted imaging, fluid-attenuated inversion recovery (FLAIR), susceptibility-weighted imaging, and postinterventional MRI were used to assess infarct morphology, lesion topology, cortical involvement, hemorrhagic transformation, cerebral microbleeds, enlarged perivascular spaces, and infarct volume. These MRI-derived features were consistently associated with epileptogenesis, particularly in acute ischemic stroke and patients undergoing mechanical thrombectomy [47,53,54].

CT remained the primary modality for ICH, with hematoma location, hemorrhage volume, and cortical extension initially incorporated into conventional prediction models before radiomics and DL enabled automated identification of imaging patterns associated with PSE [47,48]. Its widespread availability and rapid acquisition continue to support its use in clinically applicable prediction models.

Several studies also incorporated angiographic imaging during mechanical thrombectomy to evaluate collateral circulation, early venous filling, angiographic blush signs, and other procedural biomarkers associated with epilepsy risk after large-vessel occlusion stroke [51,52].

These findings suggest that vascular imaging provides complementary prognostic information beyond structural neuroimaging. Recent studies increasingly shifted from qualitative image interpretation to quantitative analysis. Radiomics enabled extraction of lesion intensity, morphology, texture, and spatial heterogeneity features, while automated segmentation and volumetric analysis provided objective measures of infarct burden and hemorrhage extent [47]. DL further advanced this approach by learning hierarchical imaging representations directly from CT images, reducing reliance on handcrafted features [48].

Overall, imaging-based prediction has evolved from visual assessment of lesion characteristics to increasingly automated quantitative analysis. Despite these advances, CT and MRI remain the foundation of current prediction models, with radiomics and DL continuing to depend on high-quality neuroimaging and standardized acquisition protocols.

### Categories of Imaging-Based Prediction Models

The included studies demonstrated a progressive evolution in methodological sophistication, ranging from conventional imaging-based statistical prediction models to radiomics, ML, DL, and explainable AI. Rather than replacing earlier approaches, contemporary AI models have largely built upon imaging biomarkers previously established through conventional statistical analyses.

The earliest generation of prediction models relied on conventional multivariable statistical techniques to quantify the relationship between structural imaging findings and subsequent seizure development. These models consistently identified infarct size, cortical involvement, lesion location, hemorrhagic transformation, middle cerebral artery territory involvement, and early PSS as important imaging-derived predictors of PSE [47,53,54]. Imaging-based prognostic scores, including SeLECT, H-SeLECT, CAVE, and the recently developed IsCHEMiA score, further translated these predictors into clinically interpretable risk stratification tools suitable for routine neurological practice [47].

Radiomics represented the first major transition toward AI-driven image analysis. Rather than relying on visually assessed imaging characteristics, radiomics-based models extracted large numbers of quantitative imaging features describing lesion texture, morphology, signal intensity, and spatial organization. These features were subsequently analyzed using ML algorithms to generate personalized risk estimates for PSE. The radiomics-clinical model developed for first-ever ICH demonstrated that combining radiomic features with conventional clinical variables significantly improved predictive performance compared with clinical predictors alone, highlighting the added value of quantitative imaging biomarkers [47].

ML approaches further expanded imaging-based prediction by integrating imaging-derived variables within flexible computational models capable of modeling complex, nonlinear relationships. Tree-based ensemble methods, SVMs, gradient boosting algorithms, and random survival forests were among the most frequently applied techniques. More recent studies also incorporated explainable AI methods, including Shapley Additive Explanations (SHAP), enabling interpretation of feature importance while maintaining high predictive performance [49]. These developments represent an important step toward improving clinician trust and facilitating future implementation of ML within stroke care.

DL constituted the most advanced category of prediction models identified in this review. Unlike conventional ML approaches that require manual feature engineering, DL architectures automatically learned hierarchical imaging representations directly from neuroimaging data. The poststroke epilepsy network (PSENet) framework exemplified this progression by using CNNs to analyze noncontrast CT images for automated prediction of PSE following ICH, thereby eliminating the need for handcrafted radiomic feature extraction [48]. Although promising, DL models remain relatively uncommon within the current literature and continue to require large annotated imaging datasets for robust development and validation.

A smaller number of studies adopted multimodal AI frameworks by integrating neuroimaging with complementary clinical or electrophysiological information. These models combined MRI-derived imaging characteristics with EEG, laboratory biomarkers, or routinely collected clinical variables to improve prediction accuracy beyond that achievable using a single data modality alone [50]. While multimodal learning demonstrated encouraging predictive performance, the increased complexity of data acquisition and integration may present additional challenges for implementation within resource-constrained health care systems.

Collectively, the evidence illustrates a gradual transition from conventional imaging-derived prediction toward increasingly sophisticated computational imaging approaches. Despite these methodological advances, conventional imaging-based prediction models remain the predominant evidence base, whereas radiomics, DL, and explainable AI represent emerging areas of investigation with comparatively limited external validation and clinical implementation.

### Computational Methods and Model Development

The computational methods used across the included studies reflected the rapid evolution of predictive modeling for PSS and PSE. Early investigations relied predominantly on conventional regression-based statistical models to quantify the relationship between imaging-derived predictors and seizure risk. Logistic regression and Cox proportional hazards regression were the most frequently applied approaches, providing interpretable estimates of the contribution of established neuroimaging biomarkers such as infarct size, cortical involvement, hemorrhagic transformation, lesion location, and early PSS to PSE development [47,53]. These models formed the basis for clinically applicable prediction scores, including SeLECT, H-SeLECT, CAVE, and the recently proposed IsCHEMiA score, which remain attractive because of their simplicity, transparency, and ease of implementation in routine clinical practice.

As larger imaging datasets became available, studies increasingly adopted ML algorithms capable of modeling complex, nonlinear relationships between imaging-derived variables and seizure outcomes. Ensemble learning techniques, including RFs, gradient boosting machines, XGBoost, and random survival forests, were among the most frequently evaluated algorithms, while SVMs were commonly used for binary classification problems [49,50]. Compared with conventional regression models, these approaches demonstrated greater flexibility in modeling interactions among imaging features and generally achieved improved predictive discrimination, particularly when combined with quantitative radiomic descriptors.

Radiomics-based investigations represented an important methodological transition by replacing subjective visual interpretation of neuroimaging with high-dimensional quantitative image analysis. Following image segmentation, hundreds of handcrafted descriptors capturing lesion intensity, morphology, texture, and spatial heterogeneity were extracted and subsequently analyzed using ML classifiers. The radiomics-clinical model developed for patients with first-ever ICH demonstrated that integrating radiomic signatures with conventional clinical predictors significantly improved model performance over clinical variables alone, highlighting the incremental value of quantitative imaging biomarkers [47]. DL approaches further reduced dependence on manual feature engineering by learning hierarchical feature representations directly from raw neuroimaging data. The PSENet framework exemplified this strategy through the application of CNNs to noncontrast CT images for automated prediction of PSE following ICH [48]. Unlike conventional radiomics pipelines, which require explicit feature extraction and selection, DL models simultaneously learned imaging features and predictive relationships during model training, thereby simplifying the analytical workflow while improving scalability.

Several recent studies also incorporated explainable AI techniques to improve model interpretability. In particular, SHAP values were used alongside random survival forests to quantify the contribution of individual imaging and clinical variables to model predictions, thereby facilitating clinical interpretation and increasing transparency for decision support [49]. Such approaches represent an important advancement toward trustworthy AI, particularly within health care environments where understanding the rationale underlying model predictions is essential for clinical adoption.

Despite substantial methodological advances, relatively few studies directly compared multiple computational approaches using identical imaging datasets, and reporting of model optimization procedures, hyperparameter tuning, calibration, and reproducibility remained inconsistent. Consequently, although contemporary ML and DL methods generally demonstrated improved predictive performance over conventional statistical models, direct comparisons between algorithms remain challenging due to heterogeneity in study populations, imaging protocols, feature engineering strategies, outcome definitions, and evaluation metrics.

### Validation Strategies

Validation strategies varied considerably across the included studies, reflecting the evolving maturity of imaging-based predictive modeling for PSS and PSE. Most studies relied on internal validation approaches, typically using random train-test splits, k-fold cross-validation, or bootstrap resampling to estimate model performance during development. Internal validation was particularly common among radiomics and ML studies because of the relatively limited availability of annotated neuroimaging datasets. Although these approaches provide useful estimates of predictive accuracy, they primarily evaluate model performance within the development population and may overestimate generalizability when applied to independent clinical settings.

The latest studies have been more inclined toward the use of independent external validation. The IsCHEMiA risk score has been externally validated by Leung et al [47] in 3 independent international cohorts from the United States, Hong Kong, and Japan. There were 2534 candidates in the validation group in addition to 1436 patients in the development group. Similarly, Galovic et al [8] examined the SeLECT model in 3 independent external cohorts where there were 1169 candidates in external validation, and the external-validation c-statistic was found to be 0.77 (95% CI 0.71‐0.82). Lin et al [57] independently validated a CT radiomics-clinical model using 2 cohorts from 2 tertiary hospitals for derivation and validation. Liu et al [64] added independent external validation to a 70:30 development split, while the independent external cohorts of 536 patients have also been used for the validation of the explainable ML model developed by Jiang et al [49]. The external C-indices ranged between 0.763 and 0.822.

However, independent external validation was rare in the literature. The vast majority of studies were validated within the same organization that generated the development data, and no external validation cohort contained patients from Africa. Therefore, questions still remain about the generalizability of these predictive models for use in African stroke populations.

Despite these improvements, external validation remained relatively uncommon across the evidence base. Most AI studies were developed using retrospective datasets collected from single institutions or regional health care systems, limiting assessment of model transportability. Furthermore, few studies reported calibration analyses, decision curve analysis, or prospective clinical evaluation, despite these being essential components for determining whether predictive models are suitable for implementation in routine neurological practice.

Reporting of model performance also demonstrated considerable heterogeneity. The AUC was the most frequently reported measure of discrimination, often accompanied by sensitivity, specificity, accuracy, and precision. Several ML studies additionally reported *F*_1_-score, positive predictive value, and negative predictive value, whereas survival-based models commonly reported the Harrell concordance index. However, inconsistent reporting of performance metrics and differing outcome definitions complicated direct quantitative comparison across studies.

Across studies reporting discrimination, AUC values ranged from 0.731 to approximately 0.99, although direct comparison was limited by differences in prediction targets, imaging modalities, algorithms, and validation strategies. The PSENet DL model reported an AUC of 0.840 (95% CI 0.792‐0.898) [48], while the CT radiomics-clinical model developed by Lin et al [57] achieved an AUC of 0.85 (95% CI 0.78‐0.92). In the multimodal ML study by Wang et al [50], validation AUCs ranged from 0.731 for RF to 0.811 for gradient boosting, whereas Liu et al [64] reported AUCs of approximately 0.99 for the best-performing tree-based models. Survival-based approaches more commonly reported concordance statistics. The IsCHEMiA model achieved an overall c-statistic of 0.848 [47], the externally validated SeLECT model achieved a c-statistic of 0.77 (95% CI 0.71‐0.82) [8], and the explainable ML model developed by Jiang et al [49] achieved external C-indices ranging from 0.763 to 0.822.

Calibration was reported less consistently than discrimination. Calibration assessment was explicitly undertaken for the IsCHEMiA model [47], the externally validated SeLECT model [8], and the explainable ML model developed by Jiang et al [49], while Brier scores were reported in some ML studies [50,64]. Strategies for addressing class imbalance were also infrequently described; Liu et al [64] explicitly used the synthetic minority over-sampling technique–edited nearest neighbors (SMOTE-ENN) to address imbalance in their large multicenter dataset. The potential for overfitting therefore remained an important concern, particularly in smaller retrospective single-center studies and models evaluated only through random train-test splitting or internal cross-validation.

Overall, the findings indicate progressive improvement in validation methodology, with increasing adoption of multicenter external validation and explainable AI. Nevertheless, prospective validation, calibration assessment, and evaluation across diverse health care systems remain limited, highlighting important priorities for future imaging-based prediction research.

### Prediction Outcomes

The included studies investigated 3 closely related but clinically distinct outcomes, namely early PSS, late PSS, and PSE. Considerable heterogeneity was observed in their operational definitions and follow-up periods. Early PSS, generally defined as seizures occurring within 7 days of stroke onset, were evaluated in a smaller proportion of studies. These studies primarily examined imaging characteristics related to acute cortical injury, infarct extent, lesion location, and hemorrhagic complications [54,62,63]. Late PSS, occurring more than 7 days after the stroke, was less frequently evaluated as an isolated prediction target. Some studies explicitly distinguished late seizures from early acute symptomatic seizures, whereas others incorporated late seizure occurrence within broader PSE outcomes [47,53,54].

PSE was the predominant prediction outcome across the included evidence, reflecting its longer-term clinical significance and association with recurrent unprovoked seizures. Conventional imaging-based models consistently identified cortical involvement, lesion location, infarct volume, hemorrhagic transformation, and stroke severity as important predictors of epilepsy development [47,53]. More recent ML and DL approaches sought to improve individualized risk estimation by integrating quantitative imaging features derived from CT, MRI, or radiomics [47,48]. Follow-up periods varied substantially across studies, ranging from predefined prediction horizons such as one year to longitudinal follow-up extending several years. This heterogeneity in outcome definitions and prediction windows complicates direct comparison of model performance across studies.

## Discussion

### Principal Findings

This scoping review mapped the global landscape of imaging-based approaches for predicting PSS and PSE, with particular emphasis on AI and its relevance to African health care settings. Across the 23 included studies, a methodological evolution was observed from conventional imaging-derived statistical models to radiomics, ML, DL, and explainable AI. Rather than representing competing approaches, these methods reflect successive stages in the development of predictive modeling, with recent AI techniques extending knowledge established by earlier studies on imaging biomarkers of epileptogenesis. At the same time, the review identified substantial heterogeneity in study design, imaging modalities, computational methods, validation strategies, and outcome definitions, limiting direct comparison between models and highlighting the need for greater methodological standardization.

One of the most consistent findings across the included studies was the central role of neuroimaging in predicting seizure-related outcomes after stroke. Earlier investigations demonstrated that cortical involvement, lesion location, infarct volume, hemorrhagic transformation, and stroke severity were independently associated with subsequent seizure development, establishing these imaging characteristics as important predictors of epileptogenesis after stroke [7,27,28,66]. These findings formed the foundation for clinically applicable prediction tools such as SeLECT, H-SeLECT, and IsCHEMiA, which combine imaging and clinical variables to estimate an individual’s risk of developing epilepsy after stroke [29,36,39]. Although these models are practical and interpretable, they depend on predefined variables and linear statistical assumptions, potentially overlooking complex interactions among neuroimaging data.

Recent advances demonstrate a shift toward computational analysis of medical images. Radiomics enabled the extraction of quantitative descriptors of lesion intensity, morphology, texture, and spatial heterogeneity, allowing imaging data to be characterized beyond conventional visual interpretation [34]. These features were subsequently incorporated into ML algorithms, improving prediction compared with models based solely on clinical or conventional imaging variables [34]. Conceptually, radiomics relies on predefined, handcrafted features extracted from medical images, whereas DL automatically learns task-relevant hierarchical features directly from image data during model training. DL further advanced this approach by learning hierarchical image representations directly from CT or MRI, without manual feature engineering. Frameworks such as PSENet illustrate the potential of end-to-end image analysis to automate prediction of PSE while reducing observer variability [67]. Similarly, explainable AI methods have begun addressing concerns regarding the interpretability of complex models by identifying the relative contribution of individual imaging and clinical variables to model predictions, thereby potentially improving transparency and supporting clinical decision-making [68].

The review also demonstrates that advances in AI have been accompanied by improvements in imaging technology. MRI was the most frequently used modality, given its ability to characterize infarct morphology, cortical involvement, lesion topology, cerebral microbleeds, and white matter abnormalities associated with epileptogenesis [29,69,70]. CT remained the preferred modality for ICH because of its widespread availability, rapid acquisition, and compatibility with radiomics and DL models [29,67]. More recently, angiographic imaging acquired during mechanical thrombectomy has emerged as an additional prognostic source, with collateral circulation and reperfusion characteristics providing complementary information for predicting epilepsy following large-vessel occlusion stroke [71,72]. These findings suggest that future prediction models are likely to integrate multiple imaging sources rather than rely on a single modality.

Although methodological sophistication has increased considerably, several limitations remain evident across the current evidence base. Most studies relied on retrospective observational datasets collected from single institutions, increasing the risk of selection bias and limiting the generalizability of the findings. External validation was performed in only a small proportion of studies, including the international validation of the IsCHEMiA model [47], while prospective evaluation and assessment of clinical impact were rarely undertaken. Furthermore, reporting of model development, calibration, hyperparameter optimization, and reproducibility was inconsistent across studies. Similar limitations have been identified in reviews of AI-based prediction models in other clinical domains, in which promising algorithmic performance has not consistently translated into routine clinical practice because of inadequate external validation and poor reporting standards. These findings highlight that improvements in predictive accuracy alone are insufficient for clinical implementation and must be accompanied by rigorous validation, transparent reporting, and assessment of clinical utility.

Another important observation was the rapid diversification of computational methods. Early studies primarily relied on regression-based analyses to quantify relationships between imaging biomarkers and seizure outcomes [7,27,28,66], whereas more recent investigations adopted ensemble learning methods, radiomics, CNNs, and explainable AI to characterize more complex imaging patterns [34,67,68]. However, relatively few studies directly compared multiple algorithms using the same datasets, making it difficult to determine whether improvements in performance reflect superior computational techniques, differences in imaging quality, larger datasets, or variations in study populations. Consequently, the current literature supports the growing potential of AI for seizure prediction but does not yet establish a single optimal modeling strategy for routine clinical use.

A key finding of this review is the absence of imaging-based AI models developed or externally validated using African neuroimaging datasets. Despite increasing global interest in AI-assisted prediction of neurological outcomes, all eligible studies originated from high-income or upper- and middle-income countries, particularly China, Japan, the United States, Austria, Germany, Italy, Argentina, and Australia [7,27-39,66-74]. This geographical imbalance extends beyond simple underrepresentation and raises important concerns regarding the transportability of existing models. AI algorithms are influenced by the characteristics of the populations on which they are trained, including disease prevalence, demographic composition, imaging protocols, health care pathways, and clinical practices. Consequently, models demonstrating excellent performance in well-resourced health care systems cannot be assumed to achieve similar accuracy when applied to African populations without appropriate external validation.

The absence of African cohorts is particularly important because stroke patterns and health care resources differ substantially across the continent. Many African hospitals primarily rely on noncontrast CT because MRI availability, specialist neuroradiology services, and advanced computational infrastructure remain limited. These differences may influence both the quality and the prevalence of imaging biomarkers associated with epileptogenesis. Furthermore, delayed presentation, limited access to reperfusion therapies, and differences in stroke etiology may alter the relationships between imaging findings and seizure risk. These factors emphasize the need for locally relevant prediction models rather than direct adoption of algorithms developed elsewhere.

Although no eligible African AI studies were identified, the review also highlights opportunities for future development. The increasing digitization of radiology services, expanding access to electronic medical records, and growing investment in AI research across Africa provide a favorable environment for collaborative model development. Importantly, several of the most promising prediction models identified in this review relied on routinely acquired CT images rather than advanced MRI sequences [29,34,67]. Since CT remains the most accessible imaging modality in many African stroke centers, CT-based radiomics and DL models may represent the most feasible starting point for future implementation. Establishing multicenter African imaging repositories would not only facilitate the development of locally relevant algorithms but also enable meaningful external validation of existing international models.

However, for the development of sustainable imaging data repositories that are multicenter and cross-continental, there needs to be a governance framework that allows privacy protection but maintains the stewardship of the institutions involved in contributing data. There must be a combination of ethical oversight, proper deidentification of digital imaging and communications in medicine (DICOM) headers and pixel data, data usage agreements, controlled access, auditing, and proper data retention and reuse procedures in place. There is also a need for common data dictionaries and quality standards of imaging so as to facilitate proper interpretations in different institutions. Equitable collaborations require clear mechanisms of data sovereignty, benefit-sharing, authorship, model ownership, and provision of results to contributing hospitals. Federated analyses could reduce the transfer of the sensitive imaging data, but do not eliminate the necessity for security, governance, and harmonization. Findable, accessible, interoperable, and reusable (FAIR) principles could enhance interoperability and reuse of data in a responsible way without full public availability [75,76].

Another important observation concerns model validation. While the methodological quality of prediction models has improved over time, external validation remains relatively uncommon. Most studies evaluated performance using internal validation techniques, such as cross-validation or random train-test splits, whereas only a few incorporated geographically independent datasets [29,68]. Internal validation provides useful evidence of model performance during development but does not demonstrate whether a model can be reliably applied in different clinical settings. The successful external validation of the IsCHEMiA score across independent international cohorts illustrates the value of evaluating prediction models beyond their development populations [29]. Nevertheless, even these validation studies were restricted to health care systems with comparable imaging infrastructure, leaving uncertainty regarding performance in resource-constrained settings.

The review also identified considerable methodological heterogeneity that limits comparison across studies. Prediction targets varied from early PSS to long-term epilepsy, while follow-up periods ranged from several months to more than 7 years. Differences were also observed in imaging modalities, feature extraction techniques, outcome definitions, evaluation metrics, and reporting standards. Such heterogeneity makes quantitative comparison difficult and partly explains the variability in reported model performance. Greater adherence to reporting frameworks for prediction modeling and AI studies would improve transparency, reproducibility, and future evidence synthesis.

The findings further suggest that advances in AI should not be viewed solely in terms of predictive accuracy. Successful clinical implementation depends equally on model interpretability, reproducibility, workflow integration, and clinician confidence. The recent incorporation of explainable AI methods represents an important step toward improving transparency by enabling clinicians to understand which imaging features contribute most strongly to individual predictions [68]. This is particularly relevant in neurological practice, where treatment decisions require clear clinical justification. Likewise, models capable of integrating into existing radiology and stroke workflows may be more likely to achieve widespread adoption than highly complex algorithms requiring extensive manual preprocessing or specialized computational resources.

Collectively, the evidence suggests that imaging-based AI is progressing from proof-of-concept research toward clinically relevant decision-support systems. However, translation into routine practice will require more than increasingly sophisticated algorithms. Future studies should prioritize prospective multicenter validation, standardized outcome definitions, transparent reporting of model development, calibration assessment, and evaluation of clinical impact. Equally important is the inclusion of underrepresented populations, particularly African stroke cohorts, to ensure that future AI models are equitable, generalizable, and capable of supporting clinical decision-making across diverse health care environments. These priorities are essential if advances in imaging-based AI are to contribute to reducing the global burden of PSE without widening existing disparities in neurological care.

### Conclusion

This scoping review provides the first comprehensive synthesis of imaging-based predictive models for PSS and PSE, with emphasis on AI methodologies and their relevance to African health care settings. The findings demonstrate a progression from conventional imaging-derived prediction models to radiomics, ML, DL, and explainable AI. These advances have improved the use of quantitative neuroimaging for individualized seizure risk prediction and highlight the growing role of AI in precision stroke care. However, substantial heterogeneity in imaging modalities, computational approaches, validation strategies, and outcome definitions limits comparison across studies and hinders clinical translation.

A key finding is the absence of imaging-based AI models developed or externally validated using African neuroimaging datasets. Although AI-assisted stroke prediction has advanced rapidly, existing models have been developed almost exclusively in high-income health care systems, leaving uncertainty regarding their applicability and generalizability to African populations. Addressing this gap requires multicenter African neuroimaging repositories, collaborative model development, and rigorous external validation using representative patient cohorts to support equitable implementation of AI in neurological care.

Future research should prioritize methodological standardization, prospective multicenter validation, transparent reporting, and clinically interpretable AI that integrates into routine stroke care. Emerging approaches, including explainable AI, transfer learning, federated learning, and multimodal predictive modeling, offer opportunities to improve model performance and clinical applicability. Ultimately, successful translation of imaging-based AI will depend on robust, transparent, and contextually relevant prediction models that support evidence-based clinical decision-making and improve outcomes for patients at risk of PSE.

### Strengths and Limitations

Although this review identified important advances in imaging-based prediction of PSS and PSE, it also highlights several limitations in the current evidence. Most included studies were retrospective and relied on datasets collected within single institutions, limiting population diversity and increasing the risk of selection bias. Sample sizes varied substantially, and relatively few studies incorporated prospective validation or independent external validation cohorts. Furthermore, differences in imaging acquisition protocols, feature engineering, AI algorithms, and outcome definitions reduced comparability between studies and prevented meaningful quantitative synthesis. These findings indicate that the field remains in an early stage of methodological maturity despite recent technological advances.

This review has several strengths. To our knowledge, it is the first to comprehensively synthesize imaging-based prediction models for PSS and PSE while explicitly examining their implications for African health care settings. Unlike previous reviews that focused primarily on epidemiology or clinical risk factors, this review evaluated imaging modalities, computational methods, validation strategies, and geographical representation. Adherence to the PRISMA-ScR reporting guideline, the JBI methodology, and prospective protocol registration further strengthens the transparency and reproducibility of the review.

Several limitations should also be acknowledged. Only English-language publications were included, which may have excluded relevant studies published in other languages. In keeping with scoping review methodology, a formal risk-of-bias assessment was not undertaken because the review aimed to map the available evidence rather than assess the certainty of individual studies. In addition, the heterogeneity of prediction targets, imaging modalities, computational approaches, and reported performance metrics limited direct comparison across studies. Finally, because no eligible African imaging-based AI studies were identified, conclusions regarding implementation in African health care settings are based on recognized evidence gaps rather than empirical evaluation.

## Supplementary material

10.2196/96828 Multimedia Appendix 1 Documentation of the search strategies.

10.2196/96828 Checklist 1 PRISMA-ScR checklist.

## Abbreviations

AUC: area under the receiver operating characteristic curve
CAVE: cortical involvement, age, volume, and early seizures
CNN: convolutional neural network
CNS: central nervous system
CT: computed tomography
DICOM: digital imaging and communications in medicine
DL: deep learning
DWI: diffusion-weighted imaging
EEG: electroencephalography
FAIR: findable, accessible, interoperable, and reusable
FLAIR: Fluid-Attenuated Inversion Recovery
H-SeLECT: hemorrhagic transformation, severity of stroke, large-artery atherosclerotic etiology
ICH: intracerebral hemorrhage
ILAE: International League Against Epilepsy
IsCHEMiA: infarct size, cortical involvement, hemorrhagic transformation, early seizures, middle cerebral artery involvement, and aged younger than 65 years
JBI: Joanna Briggs Institute
ML: machine learning
MRI: magnetic resonance imaging
OSF: Open Science Framework
PCC: population-concept-context
PRISMA-ScR: Preferred Reporting Items for Systematic Reviews and Meta-Analyses Extension for Scoping Reviews
PSE: poststroke epilepsy
PSENet: poststroke epilepsy network
PSS: poststroke seizures
RF: random forest
RNN: recurrent neural network
SeLECT: severity of stroke, large-artery atherosclerotic etiology, early seizures, cortical involvement, and the territory of the middle cerebral artery involvement
SHAP: Shapley Additive Explanations
SMOTE-ENN: synthetic minority over-sampling technique–edited nearest neighbors
SVM: support vector machine
T2-weighted: Transverse relaxation time–weighted
XGBoost: extreme gradient boosting
