# Supplementary material for: AI for Poststroke Epilepsy: Scoping Review of Imaging-Based Prediction Models and Implications for African Settings
Source: JMIR AI. 2026 Oct 16;5:e96828. doi: 10.2196/96828 (PMC13674589; doi:10.2196/96828)
Supplement: Supplementary file 1 [file ai-v5-e96828-s001.docx]

Complete Search Strategies

**Artificial Intelligence for Post-Stroke Epilepsy: A Scoping Review of Imaging-Based Prediction Models and Implications for African Settings**

**Purpose.** This appendix provides reproducible, database-specific search strategies for the updated scoping review. The same four concept blocks are retained across databases: stroke; post-stroke seizures/epilepsy; neuroimaging; and prediction/modelling. Syntax is adapted to each platform.

# 1. Master Search Logic

(stroke OR "ischemic stroke" OR "ischaemic stroke" OR "intracerebral hemorrhage" OR "intracerebral haemorrhage" OR "cerebral infarction" OR "large vessel occlusion")

AND

("post-stroke seizure" OR "post-stroke seizures" OR "poststroke seizure" OR "poststroke seizures" OR "post-stroke epilepsy" OR "poststroke epilepsy" OR "secondary epilepsy")

AND

("computed tomography" OR CT OR "magnetic resonance imaging" OR MRI OR neuroimaging OR "brain imaging" OR radiomic* OR "diffusion-weighted imaging" OR DWI OR angiograph* OR ASPECTS OR "infarct volume" OR "lesion location")

AND

(predict* OR prognos* OR model* OR "risk score" OR nomogram* OR regression OR "logistic regression" OR "Cox regression" OR "proportional hazards" OR SeLECT OR CAVE OR "artificial intelligence" OR "machine learning" OR "deep learning" OR "neural network" OR "random forest" OR "support vector machine" OR "gradient boosting" OR XGBoost OR CNN OR transformer*)

# 2. PubMed/MEDLINE

**Platform:** PubMed. **Limits:** English language; no publication-year restriction.

(
 "Stroke"[Mesh]
 OR stroke*[Title/Abstract]
 OR "ischemic stroke"[Title/Abstract]
 OR "ischaemic stroke"[Title/Abstract]
 OR "intracerebral hemorrhage"[Title/Abstract]
 OR "intracerebral haemorrhage"[Title/Abstract]
 OR "cerebral infarction"[Title/Abstract]
 OR "large vessel occlusion"[Title/Abstract]
)
AND
(
 "Seizures"[Mesh]
 OR "Epilepsy"[Mesh]
 OR "post-stroke seizure"[Title/Abstract]
 OR "post-stroke seizures"[Title/Abstract]
 OR "poststroke seizure"[Title/Abstract]
 OR "poststroke seizures"[Title/Abstract]
 OR "post-stroke epilepsy"[Title/Abstract]
 OR "poststroke epilepsy"[Title/Abstract]
 OR "secondary epilepsy"[Title/Abstract]
)
AND
(
 "Neuroimaging"[Mesh]
 OR "Tomography, X-Ray Computed"[Mesh]
 OR "Magnetic Resonance Imaging"[Mesh]
 OR "Angiography"[Mesh]
 OR neuroimaging[Title/Abstract]
 OR "computed tomography"[Title/Abstract]
 OR CT[Title/Abstract]
 OR "magnetic resonance imaging"[Title/Abstract]
 OR MRI[Title/Abstract]
 OR radiomic*[Title/Abstract]
 OR "brain imaging"[Title/Abstract]
 OR "diffusion-weighted imaging"[Title/Abstract]
 OR DWI[Title/Abstract]
 OR angiograph*[Title/Abstract]
 OR ASPECTS[Title/Abstract]
 OR "infarct volume"[Title/Abstract]
 OR "lesion location"[Title/Abstract]
)
AND
(
 predict*[Title/Abstract]
 OR prognos*[Title/Abstract]
 OR model*[Title/Abstract]
 OR "risk score"[Title/Abstract]
 OR "risk scores"[Title/Abstract]
 OR nomogram*[Title/Abstract]
 OR regression[Title/Abstract]
 OR "logistic regression"[Title/Abstract]
 OR "Cox regression"[Title/Abstract]
 OR "proportional hazards"[Title/Abstract]
 OR SeLECT[Title/Abstract]
 OR CAVE[Title/Abstract]
 OR "Artificial Intelligence"[Mesh]
 OR "Machine Learning"[Mesh]
 OR "Deep Learning"[Mesh]
 OR "artificial intelligence"[Title/Abstract]
 OR "machine learning"[Title/Abstract]
 OR "deep learning"[Title/Abstract]
 OR "neural network"[Title/Abstract]
 OR "neural networks"[Title/Abstract]
 OR "random forest"[Title/Abstract]
 OR "random forests"[Title/Abstract]
 OR "support vector machine"[Title/Abstract]
 OR "support vector machines"[Title/Abstract]
 OR "gradient boosting"[Title/Abstract]
 OR XGBoost[Title/Abstract]
 OR CNN[Title/Abstract]
 OR transformer*[Title/Abstract]
)
AND english[Language]

# 3. Embase (Embase.com)

**Platform:** Elsevier Embase.com. **Limits:** English language; no publication-year restriction.

(
 'stroke'/exp
 OR stroke*:ti,ab
 OR 'ischemic stroke':ti,ab
 OR 'ischaemic stroke':ti,ab
 OR 'intracerebral hemorrhage':ti,ab
 OR 'intracerebral haemorrhage':ti,ab
 OR 'cerebral infarction':ti,ab
 OR 'large vessel occlusion':ti,ab
)
AND
(
 'post-stroke epilepsy'/exp
 OR 'post-stroke seizure'/exp
 OR 'epilepsy'/exp
 OR 'seizure'/exp
 OR 'post-stroke seizure':ti,ab
 OR 'post-stroke seizures':ti,ab
 OR 'poststroke seizure':ti,ab
 OR 'poststroke seizures':ti,ab
 OR 'post-stroke epilepsy':ti,ab
 OR 'poststroke epilepsy':ti,ab
 OR 'secondary epilepsy':ti,ab
)
AND
(
 neuroimaging:ti,ab
 OR 'computed tomography':ti,ab
 OR ct:ti,ab
 OR 'magnetic resonance imaging':ti,ab
 OR mri:ti,ab
 OR radiomic*:ti,ab
 OR 'brain imaging':ti,ab
 OR 'diffusion-weighted imaging':ti,ab
 OR dwi:ti,ab
 OR angiograph*:ti,ab
 OR aspects:ti,ab
 OR 'infarct volume':ti,ab
 OR 'lesion location':ti,ab
)
AND
(
 predict*:ti,ab
 OR prognos*:ti,ab
 OR model*:ti,ab
 OR 'risk score':ti,ab
 OR nomogram*:ti,ab
 OR regression:ti,ab
 OR 'logistic regression':ti,ab
 OR 'cox regression':ti,ab
 OR 'proportional hazards':ti,ab
 OR select:ti,ab
 OR cave:ti,ab
 OR 'artificial intelligence':ti,ab
 OR 'machine learning':ti,ab
 OR 'deep learning':ti,ab
 OR 'neural network':ti,ab
 OR 'neural networks':ti,ab
 OR 'random forest':ti,ab
 OR 'random forests':ti,ab
 OR 'support vector machine':ti,ab
 OR 'support vector machines':ti,ab
 OR 'gradient boosting':ti,ab
 OR xgboost:ti,ab
 OR cnn:ti,ab
 OR transformer*:ti,ab
)
AND [english]/lim

# 4. Scopus

**Search field:** TITLE-ABS-KEY. **Limits:** English language; no publication-year restriction.

TITLE-ABS-KEY(
 stroke* OR "ischemic stroke" OR "ischaemic stroke" OR
 "intracerebral hemorrhage" OR "intracerebral haemorrhage" OR
 "cerebral infarction" OR "large vessel occlusion"
)
AND
TITLE-ABS-KEY(
 "post-stroke seizure" OR "post-stroke seizures" OR
 "poststroke seizure" OR "poststroke seizures" OR
 "post-stroke epilepsy" OR "poststroke epilepsy" OR
 "secondary epilepsy"
)
AND
TITLE-ABS-KEY(
 neuroimaging OR "computed tomography" OR CT OR
 "magnetic resonance imaging" OR MRI OR radiomic* OR
 "brain imaging" OR "diffusion-weighted imaging" OR DWI OR
 angiograph* OR ASPECTS OR "infarct volume" OR "lesion location"
)
AND
TITLE-ABS-KEY(
 predict* OR prognos* OR model* OR "risk score" OR nomogram* OR
 regression OR "logistic regression" OR "Cox regression" OR
 "proportional hazards" OR SeLECT OR CAVE OR
 "artificial intelligence" OR "machine learning" OR "deep learning" OR
 "neural network" OR "neural networks" OR "random forest" OR
 "random forests" OR "support vector machine" OR
 "support vector machines" OR "gradient boosting" OR XGBoost OR
 CNN OR transformer*
)

**After running:** apply Language = English.

# 5. Web of Science Core Collection

**Search field:** TS (Topic). **Limits:** English language; no publication-year restriction.

TS=(
 stroke* OR "ischemic stroke" OR "ischaemic stroke" OR
 "intracerebral hemorrhage" OR "intracerebral haemorrhage" OR
 "cerebral infarction" OR "large vessel occlusion"
)
AND
TS=(
 "post-stroke seizure" OR "post-stroke seizures" OR
 "poststroke seizure" OR "poststroke seizures" OR
 "post-stroke epilepsy" OR "poststroke epilepsy" OR
 "secondary epilepsy"
)
AND
TS=(
 neuroimaging OR "computed tomography" OR CT OR
 "magnetic resonance imaging" OR MRI OR radiomic* OR
 "brain imaging" OR "diffusion-weighted imaging" OR DWI OR
 angiograph* OR ASPECTS OR "infarct volume" OR "lesion location"
)
AND
TS=(
 predict* OR prognos* OR model* OR "risk score" OR nomogram* OR
 regression OR "logistic regression" OR "Cox regression" OR
 "proportional hazards" OR SeLECT OR CAVE OR
 "artificial intelligence" OR "machine learning" OR "deep learning" OR
 "neural network" OR "neural networks" OR "random forest" OR
 "random forests" OR "support vector machine" OR
 "support vector machines" OR "gradient boosting" OR XGBoost OR
 CNN OR transformer*
)

**After running:** apply Language = English.

# 6. IEEE Xplore

**Recommended interface:** Command Search / Advanced Search. **Limits:** English-language eligibility; no publication-year restriction.

(
 stroke OR "ischemic stroke" OR "ischaemic stroke" OR
 "intracerebral hemorrhage" OR "intracerebral haemorrhage"
)
AND
(
 "post-stroke seizure" OR "post-stroke seizures" OR
 "poststroke seizure" OR "poststroke seizures" OR
 "post-stroke epilepsy" OR "poststroke epilepsy"
)
AND
(
 neuroimaging OR "computed tomography" OR CT OR
 "magnetic resonance imaging" OR MRI OR radiomics OR
 "brain imaging" OR angiography
)
AND
(
 prediction OR predictive OR prognosis OR "risk model" OR
 "risk score" OR regression OR "artificial intelligence" OR
 "machine learning" OR "deep learning" OR "neural network" OR
 "random forest" OR "support vector machine" OR
 "gradient boosting" OR CNN OR transformer
)

# 7. Google Scholar

**Approach:** Use shorter, reproducible queries rather than the full master string. Record the exact number of results screened for each query (for example, the first 200 results), and state whether results were sorted by relevance or date.

**Query 1:** "post-stroke epilepsy" neuroimaging prediction

**Query 2:** "post-stroke epilepsy" CT MRI "machine learning"

**Query 3:** "post-stroke seizure" imaging prediction model

**Query 4:** "poststroke epilepsy" radiomics OR "deep learning"

**Screening rule:** First 200 results per query
